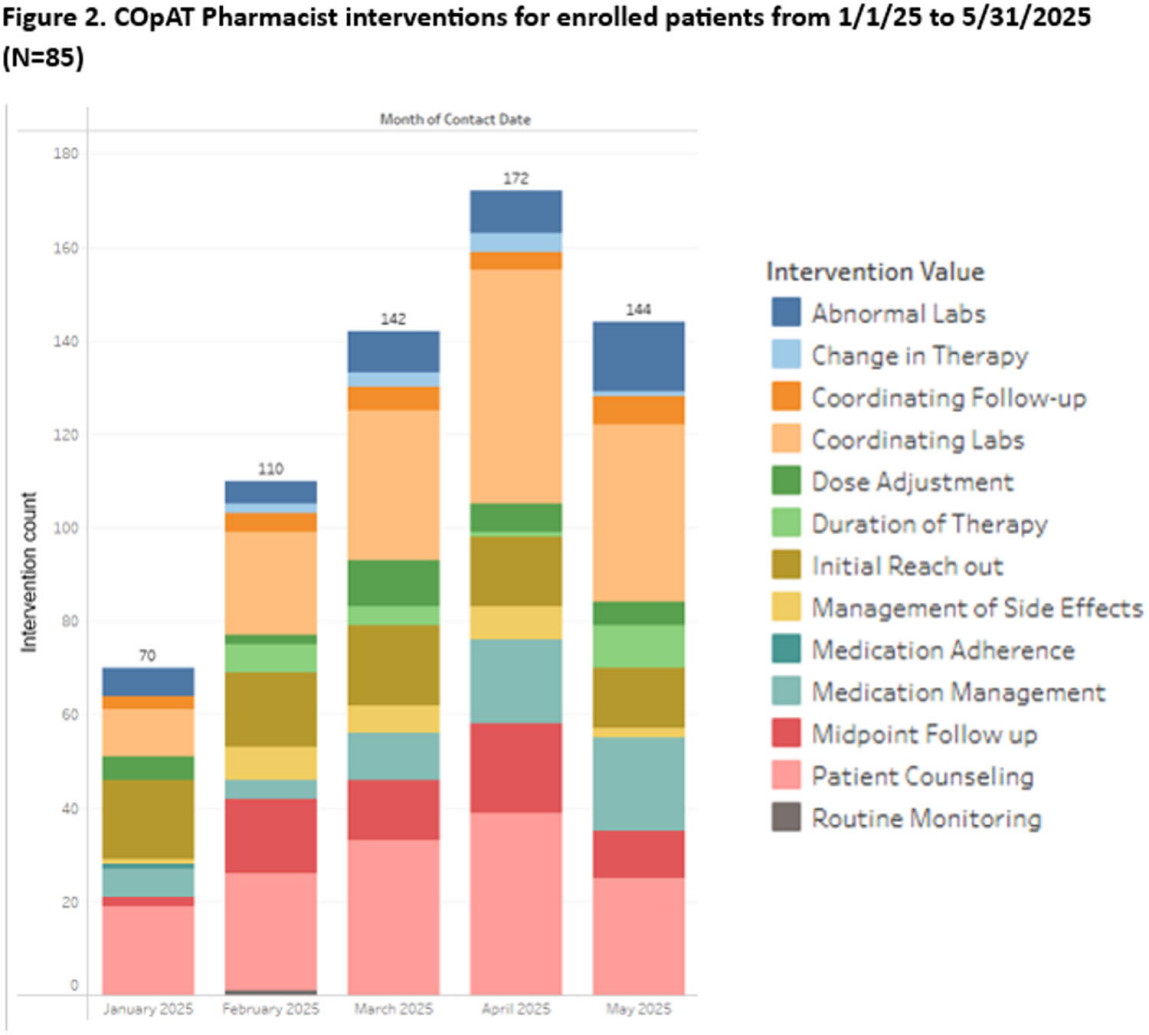# 10 Five Measles Screening Questions: Proof of Concept of Weighted Scoring System for Pediatrics

**DOI:** 10.1017/ash.2026.10464

**Published:** 2026-06-23

**Authors:** Louis Saravolatz, Tejal Gandhi, Xuping Yan, Jerod Nagel, Ashley Garrity, Shiwei Zhou, Olivia DeTroyer-Cooley, Tony Cuttitta, Jenna Keedy, James Henderson

**Affiliations:** 1 University of MIchigan; 2 University of Michigan Medical School; 3 University of Michigan Health; 4 Michigan Medicine

## Abstract

**Background:** With increasing utilization of oral antibiotics in serious infections, complex outpatient antimicrobial therapy (COpAT) programs are vital for improving patient safety and outcomes. We discuss the Michigan Medicine (MM) COpAT program, including processes, interventions, and evaluate its impact. **Methods:** Adult patients discharged from MM hospital between Jan-Oct 2025 were eligible for COpAT program enrollment if prescribed antibiotics for ≥14 days, including oral antibiotics requiring lab monitoring, long-acting lipoglycopeptides, or intravenous (IV) antibiotics administered at a hemodialysis center, subacute nursing facility, or home. Eligible diagnoses include osteomyelitis, prosthetic joint infection, septic joint, spinal hardware infection and infectious tenosynovitis. Established in October 2024, the COpAT program consists of an infectious diseases (ID) pharmacist with ID physician oversight. The pharmacist conducts a telephone-encounter within 2 business days of discharge to review antibiotic plan, lab monitoring, appropriate antibiotic administration, duration and adherence, and assess for adverse effects. The outpatient ID physician is notified of treatment concerns and lab monitoring results. Another telephone-encounter occurs 2 weeks post-discharge to re-evaluate antibiotic therapy. COpAT patient outcomes and process measures were compared to a historical cohort of patients discharged from MM on oral or IV antibiotics for an osteoarticular infection from May – August 2023. Chi-squared tests were used for categorical variables; the Mann-Whitney U test was used to compare median length of stay (LOS). **Results:** From January-October 2025, 163 patients with osteoarticular infections were enrolled in COpAT post-discharge. There were 124 patients in the historical cohort. Among COpAT patients, 79.8% (130/163) were discharged on oral antibiotics compared to 33.9% (42/124) in the historical cohort (p<0.0001, Figure 1). COpAT patients had a shorter median LOS of 6.1 days [IQR 4.2-8.7] versus 8.2 days [IQR 5.3-12.1, p<0.0001], and numerical reduction of 30-day readmission rate of 12% (19/158) versus 16.9% (22/132) in the historical cohort (p=0.258). In a subset of 85 patients enrolled from 1/1– 5/31/25, 96.5% had a COpAT telephone-encounter within 2 business days and 638 pharmacist interventions occurred with an average 7.4 interventions/patient (Figure 2). **Conclusion:** COpAT patients had significantly increased oral antibiotic utilization and shorter LOS, and numerically fewer readmissions compared to a historic cohort. The increase in oral and long-acting antibiotics, rapid post-discharge follow-up and multiple COpAT pharmacist interventions likely contributed to improved outcomes and safer transitions of care. COpAT programs should be instituted to improve patient outcomes and ensure safe use of oral and IV antibiotics in serious infections.

**Figure:**
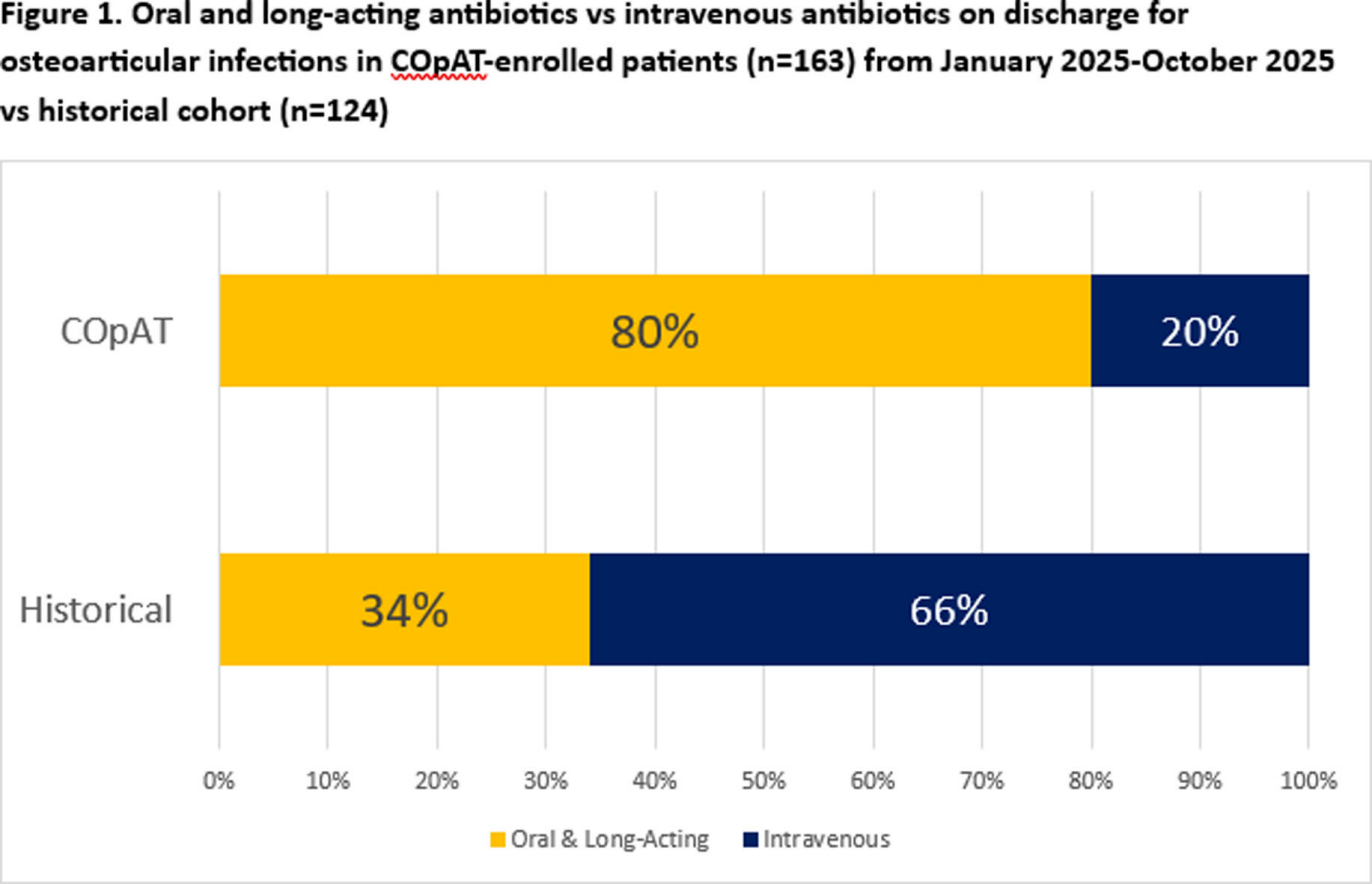

**Figure:**